# Oral Health-Related Quality of Life in People with Rare Hereditary Connective Tissue Disorders: Marfan Syndrome

**DOI:** 10.3390/ijerph15112382

**Published:** 2018-10-27

**Authors:** Marcel Hanisch, Sabrina Wiemann, Susanne Jung, Johannes Kleinheinz, Lauren Bohner

**Affiliations:** Department of Cranio-Maxillofacial Surgery, Research Unit Rare Diseases with Orofacial, Manifestations (RDOM), University Hospital Münster, Albert-Schweitzer-Campus 1, Building W 30, D-48149 Münster, Germany; s_wiem07@uni-muenster.de (S.W.); susanne.jung@ukmuenster.de (S.J.); johannes.kleinheinz@ukmuenster.de (J.K.); lauren@usp.br (L.B.)

**Keywords:** rare diseases, oral health-related quality of life, OHRQoL, Marfan, patient reported outcome, OHIP-14

## Abstract

Background: The aim of this study was to analyze data on oral health-related quality of life (OHRQoL) in people with Marfan syndrome and to obtain information on the diagnosis period, orthodontic treatment, and oral symptoms. Methods: A questionnaire was developed consisting of open questions and the standardized German version of the OHIP-14 (Oral Health Impact Profile) questionnaire for the evaluation of OHRQoL. The age of diagnosis, time period from the first signs of the disease to diagnosis, and OHIP-values were compared between male and female participants. Additionally, the OHIP-values between participants who were orthodontically treated and those who were not treated were assessed. The statistical analysis was performed using the Mann–Whitney test with a significance level at *p* = 0.05. Results: A total of 51 questionnaires were evaluated, which included 34 female and 17 male participants. Overall, 84% of respondents reported oral symptoms. Male respondents tended to diagnose the disease earlier (*p* = 0.00), with a smaller period between the first symptom and the diagnosis (*p* = 0.04). The OHIP-14 score was gender-neutral at 13.65 ± 13.53 points. Conclusion: In Marfan syndrome, many years (12.01 ± 11.61) elapse between the onset of first symptoms and correct diagnosis of the disease. People with Marfan syndrome have a worse OHRQoL than do the general population.

## 1. Introduction

In the European Union, a disease is considered “rare” if it affects less than one in 2000 people [1]. Hereditary connective tissue diseases such as Marfan syndrome are therefore classified as rare diseases. The prevalence of Marfan syndrome is 1:5000 for both genders [2,3].

Marfan syndrome is a genetic systemic connective tissue disorder with varied and combined symptoms of the heart, circulation, muscles, skeleton, eyes, and lungs [2]. The cause of most Marfan syndromes is a mutation in the fibrillin-1 gene on chromosome 15q21 [3,4]. The oral symptoms of Marfan syndrome include a narrow, highly arched palate with crowding of the teeth [5,6], dysgnathia, malocclusion [7], temporomandibular dysfunction [8], and changes in the number of teeth [9].

To date, only a few studies have been carried out on oral health-related quality of life (OHRQoL) in people with rare diseases [10], who report reduced OHRQoL. With respect to Marfan syndrome, the authors were unable to find any publications that reported on OHRQoL.

For this reason, a survey questionnaire composed of free text questions and the standardized German version of the OHIP-14 (Oral Health Impact Profile) was developed (Table S1). 

To assess oral health-related quality of life, the Oral Health Impact Profile 14 (OHIP-14) questionnaire, which measures the incidence of 14 different functional and psychosocial influences on OHRQoL [11], has proven to be effective.

The OHIP-14 questionnaire is the validated short form of the OHIP-49 and gathers information on the frequency of functional limitations, pain, psychological unease/discomfort, physical impairment, mental impairment, social impairment, and disadvantage/disability in the past month [12]. The responses are recorded on a Likert scale, and the maximum number of points (56) suggests a very high impact on the oral health-related quality of life of the patient [11].

The aim of this study was to collect and analyze data on OHRQoL in people with Marfan syndrome for the first time, and to obtain information on the diagnosis period and oral symptoms.

## 2. Methods

### 2.1. Study Design

The study was designed as an anonymous epidemiological survey to be carried out amongst people with Marfan syndrome to evaluate their OHRQoL.

For this purpose, a questionnaire was developed consisting of open questions and the standardized German version of the OHIP-14 (Oral Health Impact Profile) questionnaire for the evaluation of OHRQoL [12]. 

The OHIP-14 questionnaire was assigned standardized numerical values for each of the 14 questions: 0 = never, 1 = hardly ever, 2 = from time to time, 3 = often, and 4 = very frequently associated. Overall, a total value of 0 to a maximum of 56 points could be achieved. The higher the number, the worse the OHRQoL. The questions relate to feelings in the previous month. The questionnaire was digitally sent to a support group for Marfan syndrome (*Marfan Hilfe e.V.*), which is listed under the umbrella organization of self-help groups for chronic rare diseases in Germany, *ACHSE e.V.* (Allianz Chronisch Seltener Erkrankungen e.V.), in February 2016, and replies were accepted until February 2018.

Ethics approval from the Ethics Committee of the Medical Association of Westphalia-Lippe and Westphalian Wilhelms University Münster was obtained. (Ref. No. 2016-006-f-S). 

### 2.2. Participants

Anyone in Germany with a minimum age of 16 and affected by Marfan syndrome was eligible to participate. 

### 2.3. Data Source

In addition to age, gender and disease, potential oral symptoms, age of diagnosis, and the time period from the first signs of the disease (any Marfanoid symptom) to diagnosis as well as previous orthodontic treatment were queried, and individual OHIP values were calculated. Information on oral symptoms was translated into medical terms. 

### 2.4. Statistical Analysis

The statistical analysis was performed using the software SPSS 0.22 (IBM SPSS, Armonk, NY, USA). Descriptive data was assessed and the Shapiro–Wilk test was used to test the adherence of data to the normality curve. 

The variables “age”, “age of diagnosis”, “time period from the first signs of the disease to diagnosis”, and “OHIP-values” were statistically compared between male and female participants. Additionally, the OHIP values between participants who were orthodontically treated and those who were not treated were compared. Data were described as mean ± standard deviation. The Mann–Whitney test was used to compare groups (*p* ≤ 0.05).

## 3. Results 

### 3.1. Participants

A total of 51 questionnaires were evaluated, which included 34 female (66.67%) and 17 male (33.33%) participants. Regardless of gender, the age was 42.73 ± 14.50 years (range: 16–73). In female participants, the average age was 45.29 ± 11.90 years (range: 18–68), and male participants were on average 34.57 ± 17.58 years old (range: 16–73). 

### 3.2. Diagnosis Age and Diagnosis Period

The gender-independent average age at which a “Marfan syndrome” diagnosis was made was 17.32 ± 12.47 years (range: 8 months to 47 years). Female participants were on average 20.91 ± 12.91 years old at diagnosis (range: 3–41 years), and male participants were on average 8.52 ± 7.06 years old (range: 8 months to 47 years).

The period that elapsed from the first signs of the disease to diagnosis was 12.01 ± 11.61 years (range: 0 to 35 years) if considered independently of gender; female participants had an average of 14.05 ± 11.96 years (range: 0 to 35 years), and in males it was 4.14 ± 5.52 years (range: 0 to 19 years).

### 3.3. Oral Symptoms

A total of 50 participants made statements with respect to oral symptoms, 42 (84%) of whom described oral symptoms (29 women and 13 men), and eight (16%) reported no oral symptoms related to their disease (5 women and 3 men). With respect to oral symptoms, 38 of the respondents cited various forms of skeletal and dental anomalies including dysgnathia (n = 34), malocclusion (n = 21), high palate (n = 14), crowding (n = 14), hypodontia (n = 6), mineralization disorders of the teeth (n = 4), microdontia (n = 3), and temporomandibular dysfunctions (n = 6).

### 3.4. Orthodontic Treatment

In total, 68.75% of respondents had received orthodontic treatment (n = 33). Information from 48 participants was available. In the group who had received orthodontic treatment, 96.55% reported skeletal or dental abnormalities associated with Marfan syndrome, while 61.54% of the respondents who had not received orthodontic treatment reported skeletal or dental anomalies.

### 3.5. OHIP Values

The OHIP-14 score was gender-neutral at 13.65 ± 13.53 points, with 11.29 ± 9.87 for male patients and 14.69 ± 14.87 for female patients. When dividing the groups between “non-orthodontically treated” and “orthodontically treated”, the OHIP values were 10.15 ± 9.11 for patients who were not orthodontically treated and 15.03 ± 14.81 for those who were orthodontically treated.

### 3.6. Statistical Analysis

The descriptive statistics are described in Table 1. According to the statistical analyses, male participants tended to have received an earlier diagnosis (*p* = 0.002). However, there was no statistically significant difference between gender regarding the period from the first symptom to the diagnosis (*p* = 0.06). The main oral manifestations distributed according to gender are shown in Table 2.

**Figure 1:**
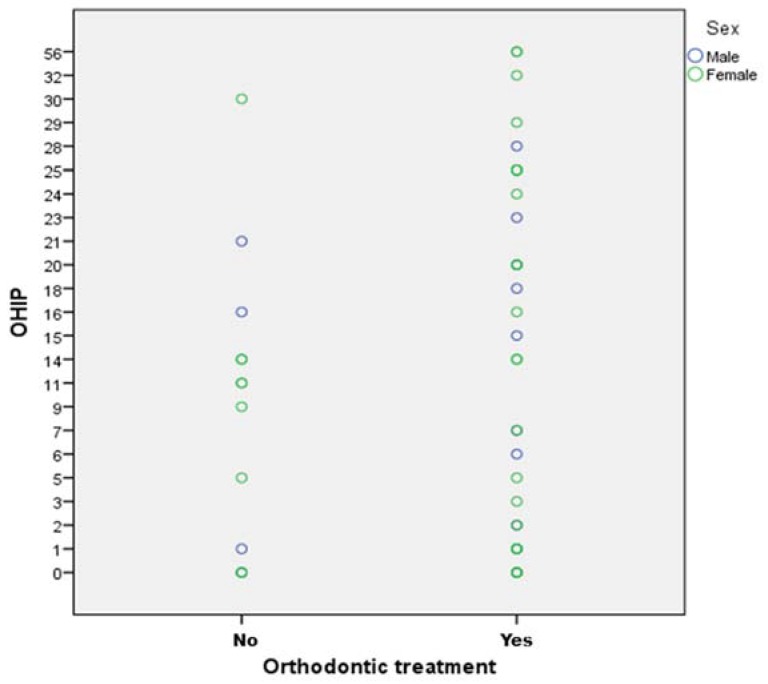
OHIP (Oral Health Impact Profile) values for “non-orthodontically treated” and “orthodontically treated” participants.

The OHIP values showed no statistically significant difference between genders (*p* = 0.693). Likewise, the orthodontic treatment did not seem to affect the OHIP-values (Figure 1), as no statistical difference was found between “orthodontically treated” and “non-orthodontically treated” participants (*p* = 0.29).

## 4. Discussion

The period that elapsed from the first signs of illness to diagnosis was 12.01 ± 11.61 years if considered independently of gender. These results confirmed that people with Marfan syndrome are affected by delays in correctly diagnosing their rare disease. On average, seven years can elapse before the correct diagnosis of a rare disease [13,14]. Due to its rarity, there are often significant delays in correctly diagnosing Marfan syndrome after the onset of first symptoms, which, on the one hand, is stressful for those affected, and, on the other hand, entails high costs due to erroneous or useless treatments [15]. Most of the respondents reported oral symptoms, therefore the dentist might be the first to be consulted by a patient with oral manifestations. Thus, dentists will be able to assist in the early diagnosis of Marfan syndrome.

The findings of the present study suggested that male participants tended to receive a diagnosis of the disease at an earlier age. These results conflicted with the study of Groth et al. (2015), who showed that the mean age of diagnosis (19 years) was not affected by gender [16]. The clinical manifestations seem to vary among those affected by the syndrome, which may lead to differences in the ages of diagnosis [17]. Nonetheless, the authors hypothesize that factors related to growth may play an important role in explaining the earlier diagnosis in males. 

Even if Marfan syndrome is equally prevalent in men and women [18], male patients with Marfan syndrome have a higher risk of an aortic event (aortic surgery or aortic dissection) than female patients [19]. This was also confirmed by Jiménez-Altayó et al. in mice [20]. It would also be of interest for future studies to study the gender differences of oral symptoms in humans in more detail.

A total of 84% of respondents with Marfan syndrome reported oral symptoms. In general, oral manifestations from rare diseases may provide an important indication of the underlying disease [5,6,7,8,9,21,22,23]. With regards to Marfan syndrome, the symptoms are commonly related to dental and facial abnormalities such as a high palate or crowding teeth. The lack of space commonly found on a high palate leads to crowding teeth, which, in turn, results in occlusion disorders (malocclusion) that could require orthodontic treatment [5,6,21]. 

The OHIP scores in the orthodontically treated group (15.03 ± 9.81) were higher than those in the group that did not report orthodontic therapy (10.15 ± 9.11), though no statistically significant difference was found between the groups (*p* = 0.29). However, it could be speculated that participants who received orthodontic treatment were affected by the severe forms of skeletal or orthodontic abnormalities associated with Marfan syndrome. This can be deduced by the fact that in this group, 96.55% reported skeletal or orthodontic anomalies, whereas only 61.54% reported anomalies in the group that did not receive orthodontic treatment.

In principle, orthodontists in particular have an important role to play in early diagnosis, as they may be consulted due to the described orthodontically-relevant symptoms. Nevertheless, the oral symptoms recorded in this study must be regarded with caution, as they have not been clinically evaluated and are based solely on information provided by the study participants. 

Previous studies have reported a high rate of orthodontic treatment for Marfan syndrome [7,21,24]. In their study, Staufenbiel et al. [21] reported that of the 62% of subjects treated orthodontically, treatment was related to high rates of skeletal and orthodontic abnormalities, as reported here. Here, too, 68.75% of respondents reported having orthodontic treatment, confirming the findings of Staufenbiel et al. [21]. In contrast, only 12% of study participants reported temporomandibular dysfunctions, which may provide important evidence of Marfan syndrome [24], while Staufenbiel reported 39.2% as having temporomandibular dysfunctions [21]. 

As opposed to patients with other types of mutations resulting in a mutant fibrillin-1 protein, a review attempting to categorize Marfan patients described more skeletal features in Marfan patients with decreased fibrillin-1 expression [25]. Future studies should analyze whetherfibrillin-1 mutations could eventually be coupled to the oral data to analyze whether oral problems occur more often in one or the other type of Marfan patients, thus providing an explanation for patients with no oral abnormalities. 

For Marfan syndrome, the OHIP-14 scores were worse for female participants than for male participants, although no statistically significant difference was found between the genders. The lack of data in the literature does not allow a direct comparison with previous studies. However, a representative study showed that in Germany an average OHIP-value of 4.09 points was determined for the standard population [26]. Standard values are used to interpret the level of impaired OHRQoL for individuals and groups of individuals compared to the degree of impaired OHRQoL in the general population [27]. It could therefore be suggested that, in Germany, people with Marfan syndrome have a worse OHRQoL than the general population.

### Limitations of the Study

The present study was carried out based on a reduced sample size, since Marfan syndrome is considered rare. Nonetheless, future investigations should aim to study even larger numbers of participants. Since not all Marfan patients have a mutation in the fibrillin-1 gene, this study did not verify which mutation the participants had. 

A second limitation of this study was the study design, which did not allow a clinical investigation of the reported symptoms. Thus, it cannot be safely assumed that all symptoms described by the respondents actually correspond to the real clinical situation. Nevertheless, this study presented data on OHRQoL in Marfan syndrome for the first time, thus enabling future studies to be based on this data.

## 5. Conclusions

People with Marfan syndrome showed an OHIP score higher than the German general population, and most of the respondents reported oral symptoms. The OHIP-14 scores were worse for female participants than for male participants. Hence, this could be an indication that additional support for dental care may be needed with regard to this rare disease.

In Marfan syndrome, many years elapse between the onset of first symptoms and the correct diagnosis of the disease, with men being younger at diagnosis and benefiting from earlier diagnosis overall than women. Therefore, dentists might be the first medical professionals to be consulted by patients with oral manifestations. Thus, dentists will be able to assist in the early diagnosis of Marfan syndrome.

## Figures and Tables

**Table 1:** Descriptive statistics.

		Mean ± SD	Median	Interquartile Range	95% CI	
					Inferior	Superior
**Age during diagnosis**	M	7.28 ± 7.06	5.00	14	0.75	13.81
	F	20.92 ± 11.61	23.00	19	16.33	25.51
**Time between diagnosis and first symptom**	M	4.14 ± 5.52	2.00	8	−0.96	9.25
	F	14.05 ± 11.96	14.00	24	9.31	18.78
**OHIP values**	M	10.57 ± 11.50	6.00	22	−0.07	21.21
	F	15.89 ± 15.83	14.00	24	9.62	22.15

M = Male; F = Female; SD = Standard deviation; 95%CI = 95% Confidence Interval.

**Table 2:** Main oral manifestations according to gender. M = Male; F = Female.

Sex	Oral Manifestations
	Dysgnathia
	Yes
**M**	11
**F**	23
**Total**	34
	High arched Palate
	Yes
**M**	1
**F**	13
**Total**	14
	Malocclusion
	Yes
**M**	5
**F**	16
**Total**	21
	Crowding
	Yes
**M**	3
**F**	11
**Total**	14
	Mineralisation disorder of the teeth
	Yes
**M**	2
**F**	2
**Total**	4
	Microdontia
	Yes
**M**	0
**F**	3
**Total**	3
	Hypodontia
	Yes
**M**	2
**F**	4
**Total**	6
	Temporomandibular disorder
	Yes
**M**	0
**F**	6
**Total**	6

## Supplementary Materials

The following are available online at http://www.mdpi.com/1660-4601/15/11/2382/s1, Table S1: Original version of the questionnaire; Table S2: Age and sex of participants, time interval from onset of the first symptoms to diagnosis, age of diagnosis, possible oral involvement, orthodontic treatment, and determined OHIP-values.
