# Supplementary material for: Oral Health-Related Quality of Life in People with Rare Hereditary Connective Tissue Disorders: Marfan Syndrome
Source: Int J Environ Res Public Health. 2018 Oct 27;15(11):2382. doi: 10.3390/ijerph15112382 (PMC6266687; doi:10.3390/ijerph15112382)
Supplement: Supplementary file 1 [file ijerph-15-02382-s001.pdf]

Supplement file:

**Hatten Sie im vergangenen Monat aufgrund von Problemen mit Ihren Zähnen, im Mundbereich oder mit Ihrem Zahnersatz ...**	**sehr oft**	**oft**	**ab und zu**	**kaum**	**nie**
....Schwierigkeiten bestimmte Worte auszusprechen?					
....das Gefühl, Ihr Geschmackssinn war beeinträchtigt?					
....den Eindruck, dass Ihr Leben ganz allgemein weniger zufriedenstellend war?					
....Schwierigkeiten zu entspannen?					
**Ist es im vergangenen Monat aufgrund von Problemen mit Ihren Zähnen, im Mundbereich oder mit Ihrem Zahnersatz vorgekommen, ...**	**sehr oft**	**oft**	**ab und zu**	**kaum**	**nie**
....dass Sie sich angespannt gefühlt haben?					
....dass Sie Ihre Mahlzeiten unterbrechen mussten?					
....dass es Ihnen unangenehm war, bestimmte Nahrungsmittel zu essen?					
....dass Sie anderen Menschen gegenüber eher reizbar gewesen sind?					
....dass es Ihnen schwergefallen ist, Ihren alltäglichen Beschäftigungen nachzugehen?					
....dass Sie vollkommen unfähig waren, etwas zu tun?					
....dass Sie sich ein wenig verlegen gefühlt haben?					
....dass Ihre Ernährung unbefriedigend gewesen ist?					
**Hatten Sie im vergangenen Monat ...**	**sehr oft**	**oft**	**ab und zu**	**kaum**	**nie**
....Schmerzen im Mundbereich?					
....ein Gefühl der Unsicherheit in Zusammenhang mit Ihren Zähnen, Ihrem Mund oder Ihrem Zahnersatz?					

OHIP-G 14 * John M, Micheelis W, Biffar R. Einflussfaktoren mundgesundheitsbezogener Lebensqualität - Validierung einer deutschen Kurzversion des Oral Health Impact Profile (OHIP-G 14). Dtsch Zahnärztl Z 2004; 59: 328-333
